# Antibiotics are too important to be left to market forces

**DOI:** 10.1093/jacamr/dlag243

**Published:** 2026-11-13

**Authors:** Elizabeth Tayler

**Affiliations:** British Society for Antimicrobial Chemotherapy, Birmingham, UK; Institute of Infection and Immunity, City St George’s University of London, London, UK

Antibiotics play a critical role underpinning health and food systems—and neither the challenges of shortages and access failures nor excess and inappropriate use will be met without intervention on the supply as well as the demand side.

Maybe this lack of focus on antibiotics, despite years of action on antimicrobial resistance, is because the price tag on the global market for generic antibiotics is only about 50 billion USD^1^—similar to that of perfume. Of course, their value—in terms of saving lives and managing the risks of invasive procedures—is much higher. Generic first-line antibiotics form the bedrock of health systems, including surgery and cancer treatments. By reducing the threat of bacterial infection they have transformed medicine and significantly increased life expectancy worldwide.

However, because they are cheap^2^ they are often inappropriately used—as a substitute for infection prevention and control, and for proper diagnosis.^3^ Because they are cheap, profit margins are low. Marketing and incentives focus on more costly antibiotics, which are often used when a cheaper drug would be more effective and safer.

The United Nations General Assembly agreed that at least 70% of all antibiotics used should come from the AWaRe Access group.^4^ AWaRe (Access, Watch Reserve and Not Recommended) is the WHO categorisation of antibiotics based on indication, resistance potential and safety. Access refers to the first-line drugs that are generally cheaper, safer and have a lower resistance potential.^5^ The market for these drugs, the bedrock of health systems, is not working. There is little interest or investment in improving this. Where there is a focus on antibiotics, it is on developing and financing new molecules—even though this will represent <1% of the total market.^6^ For the supply of the other 99%, we ‘leave it to the market’. This is in spite of the obvious failures, such as the shortages of piperacillin/tazobactam that occurred because global production was concentrated in just two factories, and one of which burned down; or the shortages of amoxicillin in many countries in the wake of COVID. In the USA, antibiotic shortages are reported 40% more often than other medications.^7⁠^

Many countries produce antibiotics in their final form, but the production of raw materials or API (Acitve Pharmaceutical Ingredient) is concentrated in China. 70% of the essential precursor for penicillin and all of the key intermediary molecule for cephalsporins is manufactured in Chinah.^8^ The disruptions now occurring in global trade mean that this is a significant risk to health systems and health security.

Some countries and regions, such as Japan and the EU, are starting to onshore the production of APIs. Building this capacity is complex and takes time. The economies of scale and the need to ensure that low- and middle-income countries are not yet left behind again mean that the manufacture of Active Pharmaceutical Ingredients should be coordinated at regional and global levels. This may require international finance and public subsidy. Developing more local pharmaceutical manufacturing capacity is a priority for many emerging economies. Where there is investment to support this from international finance, including the multilateral development banks, it should support public health objectives and clinical need. This means focusing on Access antibiotics, avoiding those that are ‘Not recommended’ by WHO, and only investing in those Watch antibiotics required in the national essential medicines list.

There should be active management of the supply chain to ensure a focus on clinical needs and not just to maximize profits. This will require better data on production and use. With the shift to digital procurement systems, mandatory standardized reporting of antibiotic use is feasible. The AWaRe system provides a framework by which it can be analysed, benchmarked and managed. Stewardship programmes have an important role in improving use, but change at scale will happen through ensuring that supply side systems and structures are properly aligned to ensure access and appropriate use of the most important medicines.
